# Supplementary material for: Evaluation of primer pairs for microbiome profiling from soils to humans within the One Health framework
Source: Mol Ecol Resour. 2020 Aug 18;20(6):1558–71. doi: 10.1111/1755-0998.13215 (PMC7693082; doi:10.1111/1755-0998.13215)
Supplement: Supplementary file 1 — Supplementary Material [file MEN-20-1558-s001.pdf]

MOLECULAR ECOLOGY RESOURCES

Supplemental Information for:

Evaluation of primer pairs for microbiome profiling from soils to humans within the One Health framework

Wasimuddin, Klaus Schlaeppi, Francesca Ronchi, Stephen L Leib, Matthias Erb, Alban Ramette

Table of Contents:

**Supplementary Table 1**	Page 2
**Supplementary Table 2**	Page 3-4
**Supplementary Table 3**	Page 5
**Supplementary Table 4**	Page 5
**Supplementary Table 5**	Page 6
**Supplementary Figure 1**	Page 7
**Supplementary Figure 2**	Page 8
**Supplementary Figure 3**	Page 9

Supplementary Table 1. Details of the samples used in the current study

Sample_ID	Source	Sample Characteristics	Sample Location	DNA Extraction Kit
S1	Soil	Soil-type1	ARA_Uetendorf	PowerSoil Pro, NucleoSpin Soil
S2	Soil	Soil-type2	Bodenacker	PowerSoil Pro, NucleoSpin Soil
S3	Soil	Soil-type3	Kurzacker	PowerSoil Pro, NucleoSpin Soil
S4	Soil	Soil-type4	Panorama	PowerSoil Pro, NucleoSpin Soil
S5	Soil	Soil-type5	Q_matte	PowerSoil Pro, NucleoSpin Soil
R1	Maize-root	Maize-root-type1	Ithaca	PowerSoil Pro, NucleoSpin Soil
R2	Maize-root	Maize-root-type2	Changins	PowerSoil Pro, NucleoSpin Soil
R3	Maize-root	Maize-root-type2	Changins	PowerSoil Pro, NucleoSpin Soil
R4	Maize-root	Maize-root-type2	Changins	PowerSoil Pro, NucleoSpin Soil
R5	Maize-root	Maize-root-type3	Reckenholz	PowerSoil Pro, NucleoSpin Soil
R6	Maize-root	Maize-root-type3	Reckenholz	PowerSoil Pro, NucleoSpin Soil
C1	Cow	Animal1	Vetsuisse	PowerSoil Pro
C2	Cow	Animal2	Vetsuisse	PowerSoil Pro
C3	Cow	Animal3	Vetsuisse	PowerSoil Pro
C4R	Cow-rumen	Animal4	Vetsuisse	PowerSoil Pro
C5R	Cow-rumen	Animal5	Vetsuisse	PowerSoil Pro
H1	Human	Male_Age-44Y	Swiss-National	PowerSoil Pro
H2	Human	Male_Age-02Y	Swiss-National	PowerSoil Pro
H3	Human	Female_Age-26Y	Swiss-National	PowerSoil Pro
H4	Human	Female_Age-43Y	Swiss-National	PowerSoil Pro
H5	Human	Male_Age-38Y	Indian-National	PowerSoil Pro
H6	Human	Female_Age-30Y	Indian-National	PowerSoil Pro
M1	Mouse	Female_ASF4-colonized	DBMR	PowerSoil Pro, QIAamp DNA Stool
M2	Mouse	Female_ASF4-colonized	DBMR	PowerSoil Pro, QIAamp DNA Stool
M3	Mouse	Female_ASF4-colonized	DBMR	PowerSoil Pro, QIAamp DNA Stool
M4	Mouse	Female_ASF4-colonized	DBMR	PowerSoil Pro, QIAamp DNA Stool
M5	Mouse	Female_ASF4-colonized	DBMR	PowerSoil Pro, QIAamp DNA Stool
MC1	Mock-community	Eight-bacterial-species	ZymoBIOMICS	NA
MC2	Mock-community	Eight-bacterial-species	ZymoBIOMICS*	NA
MC3	Mock-community	Eight-bacterial-species	ZymoBIOMICS*	NA
MC4	Mock-community	Eight-bacterial-species	ZymoBIOMICS*	NA
MC5	Mock-community	Eight-bacterial-species	ZymoBIOMICS*	NA

*ZymoBIOMICS Microbial Community DNA Standard

Supplementary Table 2. *In silico* coverage (in proportion) of primer pairs at domain and phylum level

Taxonomy level	Taxonomy for current level	Total seqs	787F-1073R	799F-1193R	515F-806R	341F-805R
Archaea	Archaea	25026	0.4914	0	0.9639	0.8359
Bacteria	Bacteria	592605	0.9691	0.8604	0.962	0.9669
Eukaryota	Eukaryota	77540	0	0	0.1663	0.0012
Bacteria	Proteobacteria	238929	0.9795	0.945	0.9799	0.9783
Bacteria	Firmicutes	149757	0.9743	0.9182	0.9662	0.964
Bacteria	Actinobacteria	60510	0.9799	0.9654	0.8796	0.9723
Bacteria	Bacteroidetes	55663	0.9669	0.9489	0.9742	0.9752
Bacteria	Acidobacteria	14534	0.9786	0.4586	0.9763	0.9788
Bacteria	Cyanobacteria	13970	0.8763	0.0088	0.9469	0.9465
Bacteria	Chloroflexi	9245	0.8852	0.2967	0.9447	0.9125
Bacteria	Planctomycetes	9014	0.9432	0.1295	0.9698	0.8776
Bacteria	Epsilonbacteraeota	5422	0.9572	0.8834	0.9817	0.9875
Bacteria	Patescibacteria	4521	0.7863	0.1694	0.8505	0.7436
Bacteria	Verrucomicrobia	4419	0.9446	0.1749	0.9615	0.9556
Bacteria	Spirochaetes	4253	0.925	0.858	0.902	0.9737
Bacteria	Tenericutes	2561	0.9559	0.4775	0.9805	0.977
Bacteria	Fusobacteria	2216	0.977	0.9666	0.9675	0.968
Bacteria	Gemmatimonadetes	2185	0.9611	0.9414	0.9593	0.9629
Bacteria	Nitrospirae	1297	0.9792	0.9545	0.9776	0.9807
Bacteria	Synergistetes	1152	0.9861	0.1615	0.9783	0.9583
Bacteria	Kiritimatiellaeota	975	0.96	0.799	0.9477	0.9631
Bacteria	Deinococcus-thermus	948	0.9631	0.9525	0.9789	0.9821
Bacteria	Armatimonadetes	752	0.9707	0.3191	0.9681	0.3218
Bacteria	Fibrobacteres	751	0.9587	0.9494	0.9614	0.9587
Bacteria	Dependentiae	580	0.981	0.8897	0.9897	0.9828
Bacteria	Atribacteria	578	0.9913	0.0969	0.9792	0.9723
Bacteria	Marinimicrobia	554	0.9838	0.9206	0.9819	0.9675
Bacteria	Omnitrophicaeota	507	0.9566	0.5089	0.9803	0.7456
Bacteria	Latescibacteria	497	0.994	0.4447	0.9819	0.9899
Bacteria	Lentisphaerae	469	0.9062	0.6269	0.9574	0.9659
Bacteria	Chlamydiae	450	0.9867	0.8178	0.9533	0.9889
Bacteria	Elusimicrobia	435	0.9724	0.9172	0.9885	0.9839
Bacteria	Rokubacteria	380	0.9737	0.0079	0.9711	0.9763
Bacteria	Brc1	380	0.9684	0.0842	0.9421	0.9684
Bacteria	Nitrospinae	364	0.9698	0.022	0.9918	0.9863
Bacteria	Aquificae	346	0.9393	0.9595	0.9595	0.9855
Bacteria	Thermotogae	303	0.1716	0.769	0.9736	0.9868
Bacteria	Cloacimonetes	301	0.9668	0.8173	0.9435	0.9568
Bacteria	Calditrichaeota	275	0.9636	0.9564	0.9455	0.9418
Bacteria	Hydrogenedentes	271	0.9779	0.1771	0.9852	0.9668
Bacteria	Halanaerobiaeota	270	0.9667	0.0444	0.9667	0.9593
Bacteria	Modulibacteria	255	0.9686	0.0392	0.9255	0.9294
Bacteria	Zixibacteria	203	0.9901	0.9606	0.9655	0.9704

Continued on page 4

Bacteria	Dadabacteria	169	0.9704	0.8817	0.9763	0.9822
Bacteria	Entotheonellaeota	168	0.9286	0.9524	0.9702	0.9643
Bacteria	Acetothermia	165	0.9697	0.2364	0.9879	0.9939
Bacteria	Fbp	155	0.1935	0.8452	0.9484	0.9613
Bacteria	Deferribacteres	134	0.8881	0.9701	0.9925	0.9851
Bacteria	Gn01	120	0.9417	0.8	0.8833	0.0583
Bacteria	Caldiserica	95	0.8947	0.9263	0.9263	0.9684
Bacteria	Ws1	93	0.9892	0.0323	0.9677	0.0323
Bacteria	Pauc34f	88	0.6364	0.7273	0.9773	0.9545
Bacteria	Margulisbacteria	86	1	0.4419	1	0.9535
Bacteria	Coprothermobacteraeota	76	0.8947	0.9474	0.9211	0.9474
Bacteria	Ta06	68	0.8971	0.6765	0.9412	0.9853
Bacteria	Aerophobetes	66	0.9848	0	0.9091	0.9242
Bacteria	Ws2	58	0.931	0.0172	0.9655	0.8621
Bacteria	Aegiribacteria	52	1	0.2692	0.9808	0.9808
Bacteria	Wps-2	51	0.9804	0.0588	0.9412	0.9412
Bacteria	Poribacteria	49	0.9796	0.0204	0.9388	0.8776
Bacteria	Lcp-89	46	0.9565	1	0.9783	1
Bacteria	Gal15	43	1	0	1	0.9767
Bacteria	Bhi80-139	41	0.9756	0.4146	0.9756	0.9756
Bacteria	Schekmanbacteria	40	0.975	0.025	1	1
Bacteria	Hydrothermae	38	0.9474	0	0.9737	0.9474
Bacteria	Fcpu426	37	0.8919	0.2432	0.9189	0.8919
Bacteria	Wor-1	34	0.9412	0.0882	0.9706	1
Bacteria	Mat-cr-m4-b07	25	1	1	0.96	1
Bacteria	Anck6	21	1	1	1	1
Bacteria	Ck-2c2-2	21	0.9524	0.7619	1	1
Bacteria	Ws4	16	1	0.0625	1	1
Bacteria	Chrysiogenetes	12	1	1	1	1
Bacteria	Dictyoglomi	11	1	0	1	1
Bacteria	Rsahf231	8	1	0	1	1
Bacteria	Uncultured	5	1	0.2	1	1
Bacteria	Fervidibacteria	4	1	1	1	1
Bacteria	Edwardsbacteria	4	1	0.25	1	0.25
Bacteria	Firestonebacteria	3	1	1	1	1
Bacteria	Calescamantes	3	1	1	1	1
Bacteria	Thermosulfidibacteraeota	3	1	1	1	1
Bacteria	Desantisbacteria	2	1	1	1	1
Bacteria	Gbs-1	2	1	1	1	1
Bacteria	Lindowbacteria	1	1	0	1	1

Supplementary Table 3. Distribution of reads at domain level and for chloroplast and mitochondria for used primer pairs

Primer pair	Bacteria	Chloroplast	Mitochondria	Archaea
787F-1073R	2,039,730	16	17340	0
799F-1193R	1,912,307	0	0	6
515F-806R	3,477,339	41592	61908	79066
341F-805R	1,560,345	49775	21685	1552

Supplementary Table 4. Summary of the best model (GLM) predicting (a) Observed species and (b) Shannon diversity according to primer pair, source, extraction kit and primer pair*source compartment interaction. Significant values (<0.05) are shown in bold.

Variables	SumsOfSqs	MeanSqs	F.Model	p
(a) Observed species				
Primerpair	31604	10535	8.0362	**<0.001**
Source compartment	810819	162164	123.7055	**<0.001**
Extraction kit	49	49	0.0376	0.846
Primer pair*Source compartment	26790	1786	1.3624	0.171
(b) Shannon				
Primerpair	4.848	1.616	11.2825	**<0.001**
Source compartment	181.966	36.393	254.0917	**<0.001**
Extraction kit	0.25	0.25	1.7454	0.188
Primer pair*Source compartment	3.805	0.254	1.771	**0.042**

Supplementary Table 5. Summary of the best model (GLM) predicting (a) Observed species and (b) Shannon diversity according to primer pair, source, extraction kit and primer pair*source compartment interaction. Significant values (<0.05) are shown in bold.

Variables	SumsOfSqs	MeanSqs	F.Model	R^2	p
(a) Jaccard					
Primerpair	2.025	0.675	3.513	0.02464	**0.001**
Source compartment	39.965	7.9931	41.597	0.48633	**0.001**
Extraction kit	0.319	0.3188	1.659	0.00388	0.052
Primer pair*Source compartment	8.739	0.5826	3.032	0.10635	**0.001**
Residuals	31.129	0.1922		0.3788	
Total	82.178			1	
(b) Bray-Curtis					
Primerpair	1.717	0.5722	4.401	0.02196	**0.001**
Source compartment	48.84	9.7679	75.124	0.62468	**0.001**
Extraction kit	0.192	0.1924	1.48	0.00246	0.142
Primer pair*Source compartment	6.371	0.4247	3.267	0.08149	**0.001**
Residuals	21.064	0.13		0.26942	
Total	78.184			1	

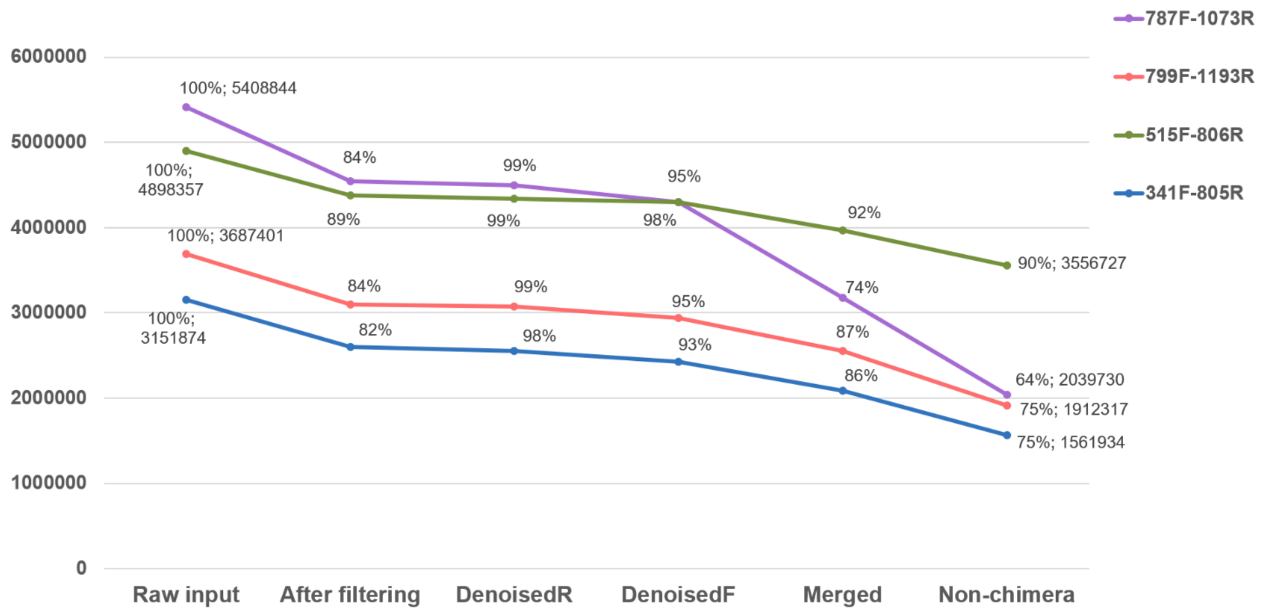

Supplementary Figure 1. Distribution of reads at different stages of amplicon processing pipeline for used primer pairs

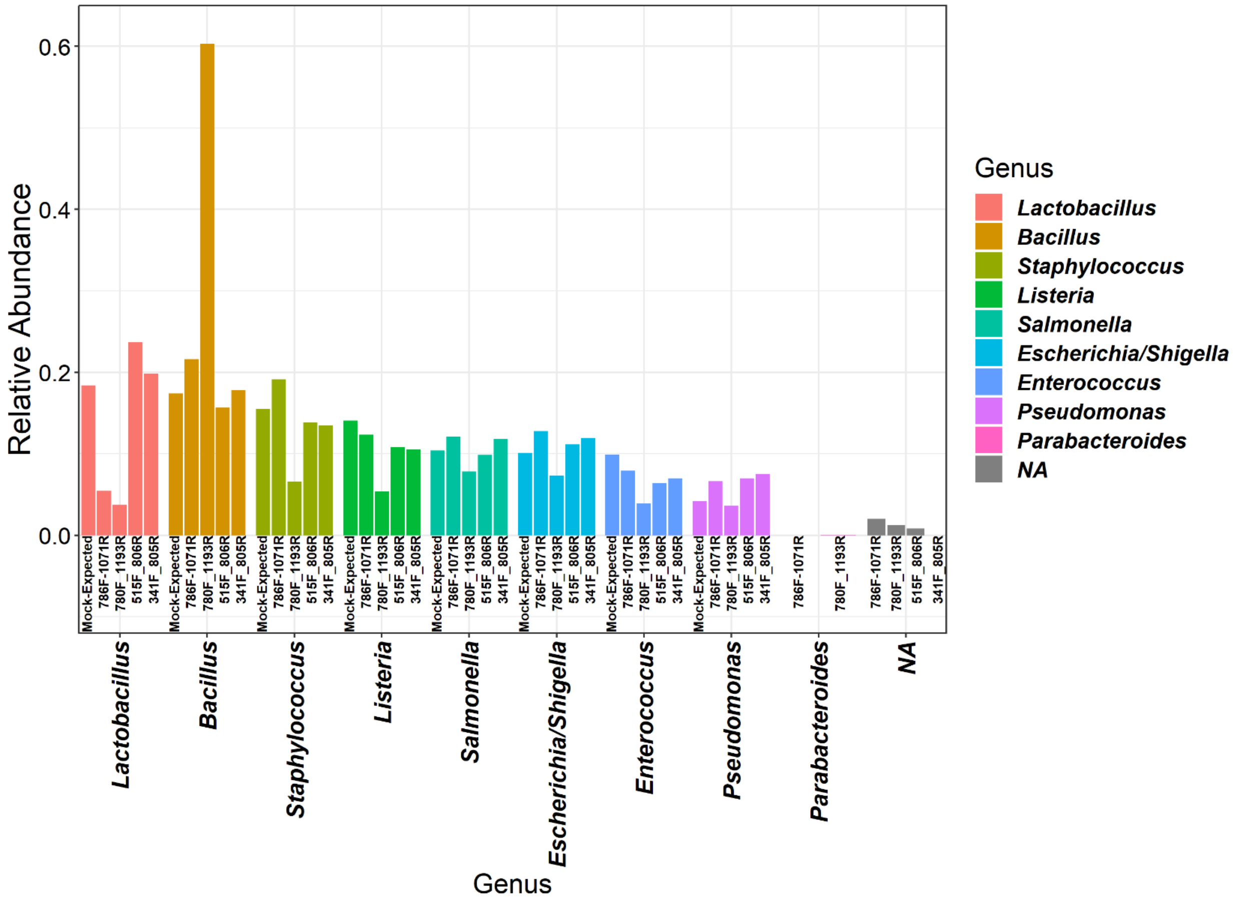

Supplementary Figure 2. Bar plots showing expected mock community, mock community amplified by four primer pairs at genus level. NA represents unassigned taxa at genus level

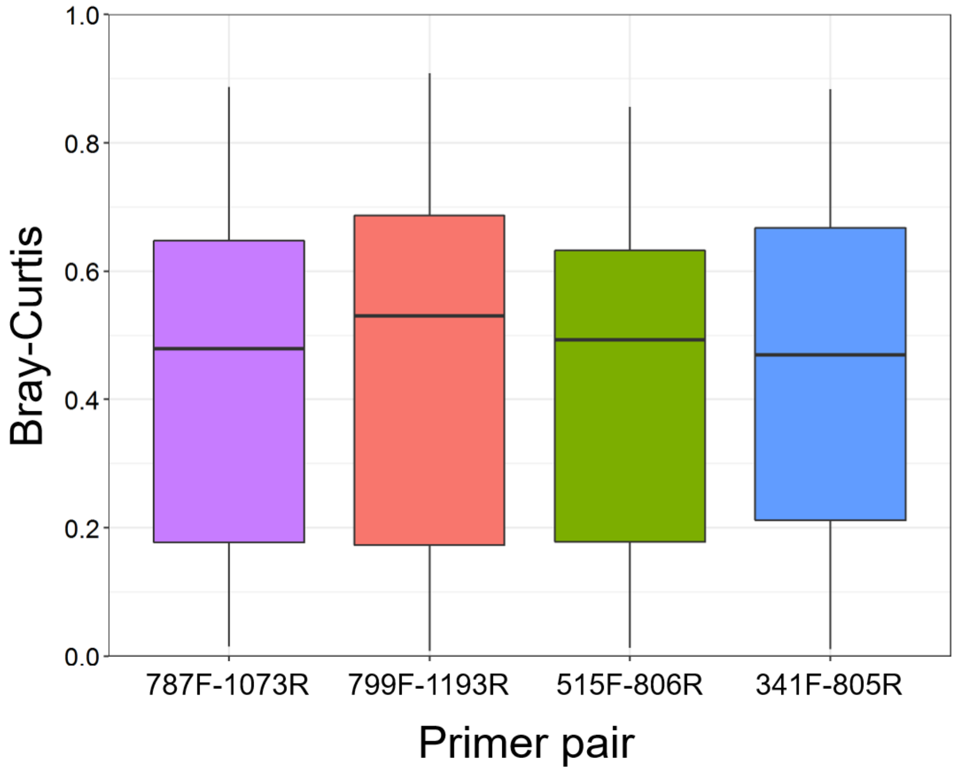

Supplementary Figure 3. Box plots showing average beta diversity based on Bray-Curtis metric for each prime pair. No significant difference in average beta diversity values was observed among primer pairs
